# Dr. Edwin C. Reames

**Published:** 1913-12

**Authors:** 


Dr. Edwin C. Reames, Buffalo, 1895, died at his home in Canastota, September 25, aged 43, of nephritis.

